# Nano-structured smart hydrogels with rapid response and high elasticity

**DOI:** 10.1038/ncomms3226

**Published:** 2013-07-31

**Authors:** Lie-Wen Xia, Rui Xie, Xiao-Jie Ju, Wei Wang, Qianming Chen, Liang-Yin Chu

**Affiliations:** 1 School of Chemical Engineering, Sichuan University, Chengdu, Sichuan 610065, China; 2 State Key Laboratory of Oral Diseases, Sichuan University, Chengdu, Sichuan 610041, China; 3 State Key Laboratory of Polymer Materials Engineering, Sichuan University, Chengdu, Sichuan 610065, China; 4 Collaborative Innovation Center for Biomaterials Science and Technology, Sichuan University, Chengdu, Sichuan 610065, China; 5 Present address: School of Chemistry, Leshan Normal University, Leshan, Sichuan 614004, China

## Abstract

Smart hydrogels, or stimuli-responsive hydrogels, are three-dimensional networks composed of crosslinked hydrophilic polymer chains that are able to dramatically change their volume and other properties in response to environmental stimuli such as temperature, pH and certain chemicals. Rapid and significant response to environmental stimuli and high elasticity are critical for the versatility of such smart hydrogels. Here we report the synthesis of smart hydrogels which are rapidly responsive, highly swellable and stretchable, by constructing a nano-structured architecture with activated nanogels as nano-crosslinkers. The nano-structured smart hydrogels show very significant and rapid stimuli-responsive characteristics, as well as highly elastic properties to sustain high compressions, resist slicing and withstand high level of deformation, such as bending, twisting and extensive stretching. Because of the concurrent rapid and significant stimuli-response and high elasticity, these nano-structured smart hydrogels may expand the scope of hydrogel applications, and provide enhanced performance in their applications.

## Abstract (web summary)

Smart hydrogels are 3D networks composed of cross-linked polymer chains that can alter their shape and properties in response to environmental stimuli. Xia *et al.* demonstrate a smart hydrogel with rapid response and high elasticity, due to its nano-scale structure.

## Graphical abstract

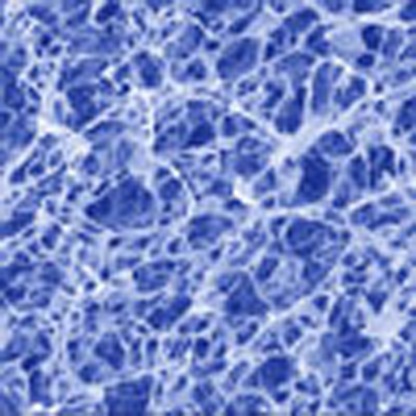

Smart hydrogels, which can dramatically change their volume and other properties in response to environmental stimuli such as temperature, pH and certain chemicals, are having an increasingly important role in myriad applications, including smart actuators for chemical valves^1^, optical systems^2^, artificial ‘muscles’^3^,^4^, soft biomimetic machines^5^ and ‘on/off’ switches for chemical reactions^6^,^7^, as well as scaffolds for tissue engineering^8^,^9^, vehicles for drug delivery^8^,^9^,^10^ and matrices for bioseparation^9^,^11^. Rapid and significant response to environmental stimuli and high elasticity are critical for the versatility of such smart hydrogels, because these attributes ensure instantaneous and remarkable feedback after receiving environmental signals and high extensibility and deformability to tolerate external forces. However, conventional hydrogels chemically crosslinked by small molecules always present slow response rate and poor elasticity, which severely limit the scope of hydrogel applications. For example, as a kind of typical thermo-responsive smart hydrogels that can undergo a reversible volume phase transition near the lower critical solution temperature (LCST, ~32 °C), normal poly(*N*-isopropylacrylamide) (PNIPAM) hydrogels crosslinked by chemical crosslinkers such as *N*,*N*-methylenebisacrylamide (MBA) are always elastically poor and shrink/swell slowly upon heating/cooling across the LCST. To overcome these limitations, intense efforts have been devoted to synthesizing hydrogels with either improved response properties^12^,^13^,^14^,^15^,^16^,^17^ or improved elastic properties^18^,^19^,^20^,^21^,^22^. To improve the response rate of PNIPAM-based thermo-responsive hydrogels, several strategies have been developed by grafting side chains onto the networks to generate comb-type grafted hydrogels^12^,^13^, generating porous structures inside hydrogels^14^,^15^ or introducing micellar structures for water pathways in hydrogels^16^,^17^. All these strategies can significantly improve the response rate of hydrogels, but they cannot help to improve the elastic property. Several other strategies have been developed to improve the elasticity of hydrogels^18^, including generating double-network hydrogels consist of two interpenetrating networks^19^,^20^, or using exfoliated clay nanoparticles as crosslinkers for synthesizing nanocomposite hydrogels^21^,^22^,^23^. The double-network hydrogels could be highly stretchable, but the stimuli-sensitivities for each polymer in the interpenetrating networks become seriously impaired because of interactions between the polymers. The nanocomposite hydrogels with clay crosslinkers are very elastic and have much better stimuli-responsive property than normal hydrogels; however, their thermo-responsive equilibrium swelling ratio is still limited, for example, the thermo-responsive equilibrium swelling ratio in responding to temperature changing from 50 °C to 20 °C is typically<3,200% (refs 21, 22). More recently, macromolecular microspheres have been utilized as building blocks or covalent crosslinkers to form either rapidly responsive hydrogels^24^,^25^,^26^,^27^ or highly elastic hydrogels^28^,^29^. However, all the macromolecular microspheres used for synthesizing microsphere-composited hydrogels are as large as sub-micron or even micron in diameter, much larger than the clay nanoparticles^22^. This might be an obstacle for the microsphere-composited hydrogels to simultaneously have large responsive swelling ratio, quick response rate and high elasticity. Up to now, the fabrication of smart hydrogels with large responsive swelling ratio, rapid response rate and high elasticity still remains a challenge.

Here we report a simple method to synthesize smart hydrogels with rapidly responsive, highly swellable and stretchable properties by constructing nano-structured architecture with controllable activated nanogels (ANGs) as nano-crosslinkers. The ANGs are responsive nanoparticles with active groups, and <60 nm in hydrodynamic diameter. Our nano-structured smart hydrogels show extraordinary large responsive swelling ratio, rapid response rate and high elasticity. The thermo-responsive swelling ratio of our nano-structured hydrogels (NSG) in responding to temperature changing from 45 °C to 15 °C is as high as 8,000%, and the characteristic time of gel shrinking from fully swollen state to fully collapsed state upon heating from 25 °C to 55 °C for a NSG hydrogel with characteristic dimension of >22 mm is only ~6 min. Meanwhile, our NSG hydrogels are elastic enough to withstand high compressions, slicing with a blade and high level of deformations such as bending, twisting, knotting and extensive stretching. Our NSG hydrogels can be stretched beyond 1,800% their initial length at room temperature (25 °C). Importantly, the large responsive swelling ratio, rapid response rate and high elasticity of our NSG hydrogels can be obtained simultaneously without any conflict.

## Results

### Fabrication of nano-structured smart hydrogels

**Figure 1:**
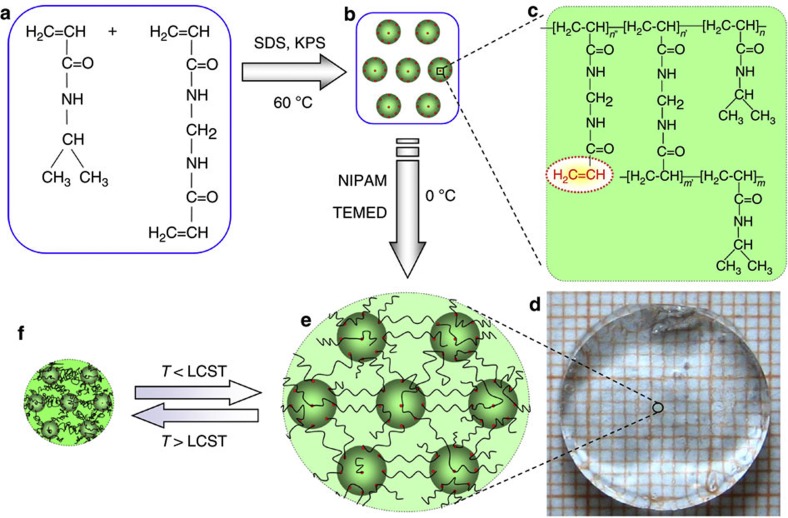
Hydrogel schematics with nano-structured architecture. (**a**) Chemical structure of NIPAM and MBA. (**b**) ANGs bearing unsaturated double bonds fabricated by precipitation polymerization of NIPAM and MBA, which is initiated by KPS in the presence of amphiphilic SDS. (**c**) Chemical structure of the ANG showing the unsaturated double bonds from the unreacted parts of MBA. (**d**) Optical image showing the NSG, which is fabricated from the ANGs and NIPAM with TEMED as an accelerator at 0 °C, is transparent at temperature below the LCST. (**e**,**f**) Schematics of the NSG hydrogel with nano-structured architecture in the swollen state at temperature below the LCST (**e**) and in the shrunken state at temperature above the LCST (**f**).

**Figure 2:**
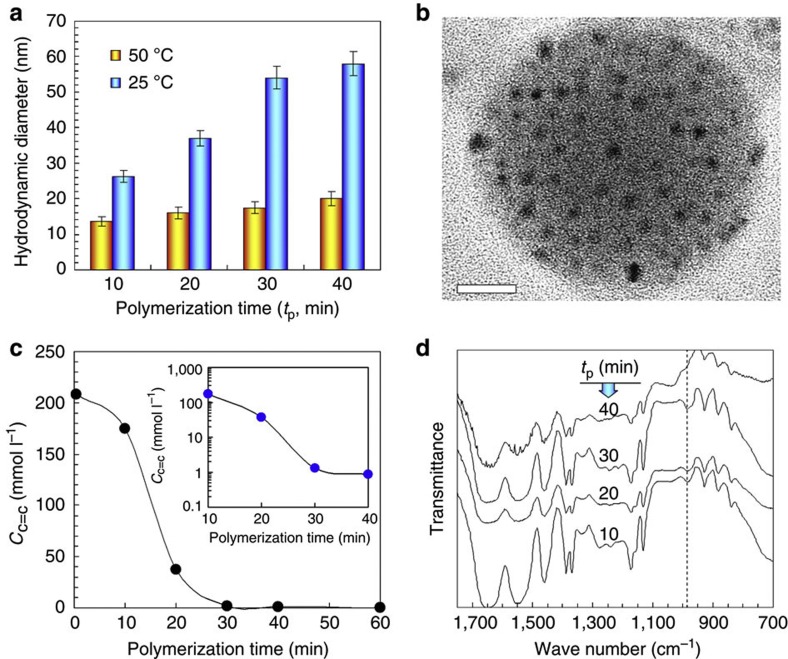
Fabrication of ANG nanogels bearing unsaturated double bonds. (**a**) Effects of polymerization time and temperature on the average hydrodynamic diameters of ANG nanogels in water detected by dynamic light scattering. (**b**) TEM micrograph of dried nanogels (ANG30), in which the randomly distributed dark dots with average diameter of ~6 nm in the shadow area are the ANG nanogels. Scale bar, 20 nm. (**c**) Effect of polymerization time on the concentration of unsaturated double bonds in the ANG dispersions (*C*_C=C_). (**d**) FT-IR spectra of thoroughly dialyzed ANG nanogels prepared with polymerization time (*t*_p_) of 10, 20, 30 and 40 min respectively, in which the peak at 981 cm^−1^ refers to unsaturated double bonds.

The fabrication procedure for our NSGs is very simple and controllable (Fig. 1). First, controllable ANG bearing unsaturated double bonds are fabricated by precipitation polymerization of NIPAM and MBA at 60 °C, which is initiated by potassium persulfate (KPS) in the presence of SDS (Fig. 1a,b). Usually, it is difficult to fabricate nanogels of <100 nm with precipitation polymerization. In the precipitation polymerization, poly(NIPAM-*co*-MBA) macromolecules precipitate from aqueous solution at temperature higher than the LCST of PNIPAM and form gel particles. We use the amphiphilic SDS molecules to prolong the nucleation and precipitation process of hydrophobic poly(NIPAM-*co*-MBA) macromolecules and hinder the growth of gel particles. As a result, thermo-responsive PNIPAM nanogels with hydrodynamic diameter of <100 nm are successfully synthesized. For nanogels prepared with polymerization time periods of 20 min and 40 min, their average hydrodynamic diameters are respectively 16 nm and 20 nm at 50 °C (Supplementary Fig. S1). With increasing the polymerization time, the nanogel size increases slightly and the thermo-responsive volume change property is also improved slightly (Fig. 2a). The TEM micrograph of dried nanogels prepared with polymerization time of 30 min shows that the average diameter is ~6 nm (Fig. 2b), which is smaller than that in water at 50 °C (Fig. 2a) due to the complete dehydration. The attachment of amphiphilic molecules on the surface of PNIPAM gel particles also restrains the diffusion of monomer molecules and radicals into and out of the PNIPAM gel particles. Thus, unsaturated double bonds from the unreacted parts of MBA molecules (Fig. 1c) are reserved controllably in the gel particles because it is difficult for them to meet radicals to initiate polymerization. The concentration of unsaturated double bonds in the ANG nanogel dispersions decreases rapidly with increasing the polymerization time (Fig. 2c). If the polymerization time is as long as 60 min, no unsaturated double bonds can be found in the ANG nanogel dispersions anymore. However, when the polymerization time is not longer than 40 min, ample unsaturated double bonds exist in the thoroughly dialyzed PNIPAM nanogels (Fig. 2d). It has been confirmed that the consumption of MBA is much faster than that of NIPAM in the PNIPAM gel particle formation by precipitation polymerization^30^. Therefore, the longer the precipitation polymerization, the fewer the unsaturated double bonds left from the MBA molecules in the solution. The unsaturated double bonds in PNIPAM nanogels are essentially important for the formation of NSG hydrogel networks. So, ANGs bearing ample unsaturated double bonds are successfully fabricated with polymerization time periods of 10, 20, 30 and 40 min, which are labelled as ANG10, ANG20, ANG30 and ANG40 respectively.

Second, the NSG hydrogels are fabricated from the ANG nanogels and NIPAM monomer with *N*,*N*,*N*′,*N*′-tetramethylethylenediamine (TEMED) as an accelerator at 0 °C (Fig. 1). NIPAM is dissolved in the aqueous dispersion of as-prepared nanogels. When TEMED meets the initiator KPS left in the nanogel dispersion, a redox reaction starts to polymerize the NIPAM monomers. PNIPAM radicals generated in the aqueous dispersion initiate NIPAM monomers, and thus they propagate to meet and initiate unsaturated double bonds in the ANG nanogels to form longer PNIPAM radicals. These PNIPAM radicals propagate to form grafting PNIPAM radicals by initiating NIPAM monomers in the aqueous solution. When the grafting PNIPAM radicals meet and initiate unsaturated double bonds in neighbouring ANG nanogels, PNIPAM bridge chains are formed to connect the neighbouring ANG nanogels. If some grafting PNIPAM radicals are terminated by other free radicals, they become dangling grafting PNIPAM chains on the nanogels. Finally, NSGs with PNIPAM nanogels as nano-crosslinkers and PNIPAM chains as polymer networks are constructed (Fig. 1e). NSG hydrogels are synthesized by varying the polymerization time of ANG nanogels, the NIPAM molar concentration and the ANG nanogel content. The resultant NSG hydrogels are designated as ‘NSG*t*_p_-*C*_N_’, in which ‘*t*_p_’ indicating the polymerization time of ANG nanogels and ‘*C*_N_’ representing the NIPAM molar concentration in the hydrogel preparation (Table 1). For example, a code of NSG40-1.5 means that the NSG hydrogel is prepared with ANG40 nanogels and 1.5 mol l^−1^ NIPAM concentration, and so on. Especially, a suffix ‘0.5C’ is added to the code of the NSG hydrogels when the ANG nanogel content is reduced to 50% (Table 1). As a reference, a normal hydrogel (NG) is also prepared with MBA molecules as crosslinkers, and labelled as NG2-1.5.

**Figure 3:**
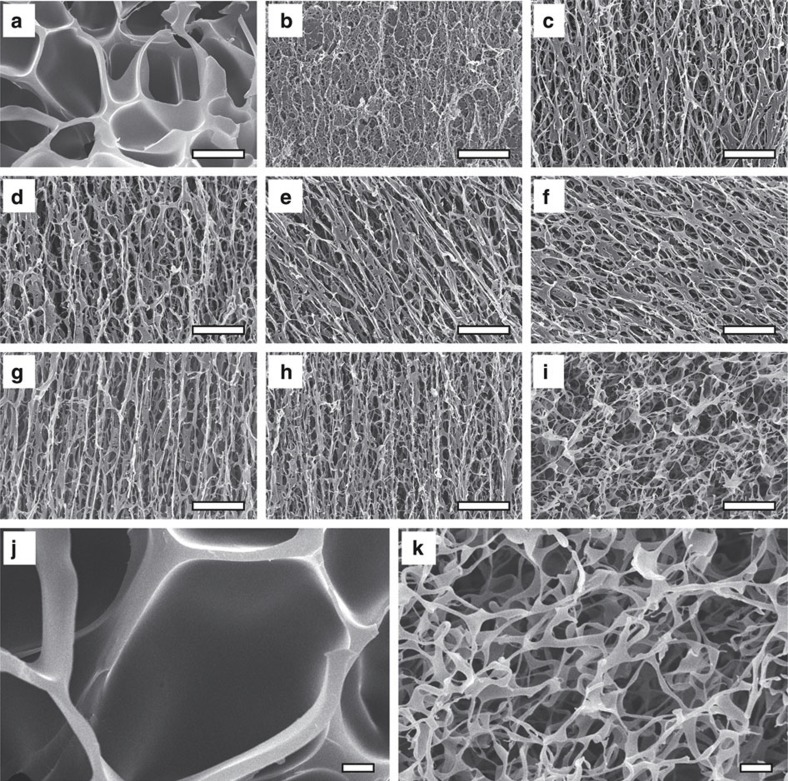
SEM images of NG and NSG hydrogels. (**a**) NG2-1.5; (**b**) NSG10-1.5; (**c**) NSG20-1.5; (**d**) NSG30-1.5; (**e**) NSG40-1.5; (**f**) NSG40-1.0; (**g**) NSG40-0.5; (**h**) NSG40-0.25; (**i**) NSG40-1.5-0.5C; (**j**,**k**) partially enlarged views of (**a**,**i**). Scale bars are 5 μm in (**a**–**i**) and 1 μm in (**j**,**k**).

The as-prepared NSG hydrogels are always transparent at temperatures below the LCST of PNIPAM, regardless of the polymerization time of ANG nanogels, the NIPAM molar concentration and the ANG nanogel content (Fig. 1d). This phenomenon indicates that the nanogels are homogeneously distributed in the NSG hydrogel networks. Fourier transform infrared spectroscope (FT-IR) characterization results show that, SDS is not found in the NSG hydrogels after being fully washed by water, and unsaturated double bonds exist in thoroughly dialyzed ANG nanogels clearly but not in NSG hydrogels anymore (Supplementary Fig. S2). Scanning electron microscope (SEM) micrographs show that the microstructures of freeze-dried NSG hydrogels are significantly different from that of freeze-dried NG hydrogel (Fig. 3). The freeze-dried NG hydrogel shows honeycomb-like structure with nearly dense cell walls as usual (Fig. 3a,j), but the freeze-dried NSG hydrogels show heterogeneous crosslinked mesh-like structures (Fig. 3b–i,k). The micropores within the NG hydrogel after freeze-drying result from the ice crystals presented in the swollen NG hydrogel acting as a template for pore generation^31^,^32^. As water inside the swollen NG hydrogel is dispersed in the crosslinked polymeric network, the ice crystals rapidly form within the network of NG hydrogel upon immersing the sample into liquid nitrogen. The frozen NG hydrogel is dried by sublimation of ice crystals under vacuum at a temperature below the ice freezing point; as a result, micropores are formed within the NG hydrogel networks, exhibiting a honeycomb-like structure with nearly dense cell walls. However, for the NSG hydrogels, because of the elastic property of the grafted long PNIPAM chains between the nanogels, water inside the swollen NSG hydrogels exists in a more interconnected state at the microcosmic level than that inside the NG hydrogel. Consequently, the ice-templated porous structures in NSG hydrogels show heterogeneous crosslinked mesh-like structures. Although it seems that the micropores of NSG hydrogels are much smaller than that of the NG hydrogel, the micropores inside the NSG hydrogels are all interconnected. Such porous structures with interconnected micropores inside the NSG hydrogels provide efficient channels for rapid water transport, which is beneficial to the rapid response of hydrogels. Compared to NSG20-1.5, NSG30-1.5 and NSG40-1.5 hydrogels, NSG10-1.5 hydrogel is significantly different, with a much denser network structure (Fig. 3b), due to the much larger amount of unsaturated double bonds in the ANG10 nanogel dispersion (Fig. 2c). The microstructures of freeze-dried NSG hydrogels do not change remarkably with varying the NIPAM molar concentration (Fig. 3e–h). With reduced ANG nanogel content, the NSG40-1.5-0.5C hydrogel becomes less crosslinked, and the freeze-dried sample is featured with much larger internal pores (Fig. 3i,k).

### Responsive properties of nano-structured smart hydrogels

**Figure 4:**
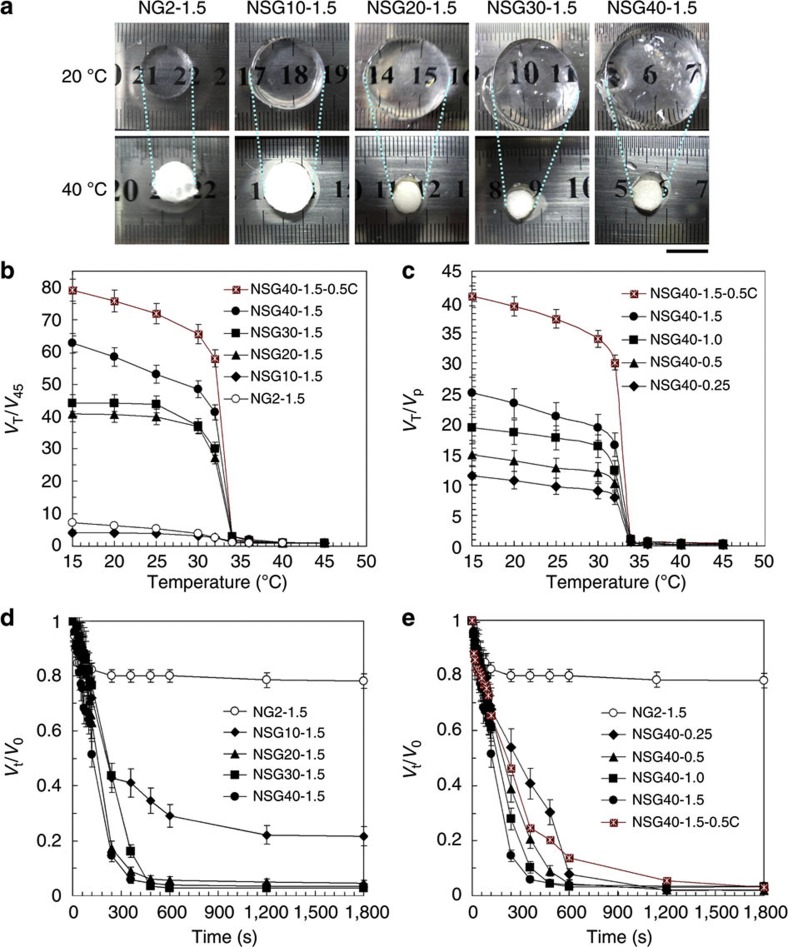
Thermo-response of NSGs. (**a**) Optical images showing the NSG and NG hydrogel samples in swollen states at 20 °C (<LCST) and in shrunken states at 40 °C (>LCST) in pure water. Scale bar, 10 mm. (**b**,**c**) Temperature dependence of the equilibrium swelling ratios of NSG and NG hydrogels, in which *V*_T_ and *V*_45_ are the equilibrium volumes of hydrogels at *T* °C and 45 °C respectively, and *V*_p_ is the initial volume of hydrogels in the preparation tube. (**d**,**e**) Dynamic volume-deswelling behaviours of NSG and NG hydrogels after the environmental temperature jumping abruptly from 25 °C to 55 °C, in which *V*_t_ and *V*_0_ are the volumes of hydrogels at time *t* and at beginning (*t*=0, equilibrated in water at 25 °C) respectively.

Our NSGs show significant responsive swelling ratio and rapid response rate in responding to environmental temperature change across the LCST (Fig. 4). All the NSG hydrogels have very large thermo-responsive equilibrium swelling ratio except for NSG10-1.5 (Fig. 4a,b). It is well known that the thermo-responsive equilibrium swelling ratio decreases on increasing the crosslinkage^33^. Owing to the large amount of unsaturated double bonds in ANG10 nanogel dispersion and high concentration of NIPAM monomer, the NSG10-1.5 hydrogel is highly crosslinked; therefore, the thermo-responsive equilibrium swelling ratio is low. On increasing the polymerization time of ANG nanogels but keeping the NIPAM concentration constant, the crosslinkage of NSG hydrogels decreases due to the reduced unsaturated double bonds in ANG nanogel dispersions; as a result, the thermo-responsive equilibrium swelling ratio of NSG hydrogels increases (Fig. 4b). On keeping the polymerization time of ANG nanogels constant but increasing the NIPAM concentration, the number of crosslinking points per unit volume of NSG hydrogels keeps the same but the PNIPAM bridge chains connecting the nanogels become longer because NIPAM monomers are supplied more sufficiently; as a result, the thermo-responsive equilibrium swelling ratio of NSG hydrogels also increases (Fig. 4c). On decreasing the concentration of ANG nanogel dispersion, the thermo-responsive equilibrium swelling ratio of NSG hydrogels increases further. For the NSG40-1.5-0.5C, the thermo-responsive equilibrium swelling ratio in responding to temperature changing from 45 °C to 15 °C is as high as 8,000%, which is 10 times larger than that of the normal hydrogel NG2-1.5, and also much larger than that of nano-clay-composited hydrogels (typically <3,200%)^21^,^22^ and microsphere-composited hydrogels (typically <4,000%)^24^,^25^,^26^,^27^. In fact, the generated NSG hydrogels are heterogeneous three-dimensional (3D) networks composed of PNIPAM chains crosslinked by PNIPAM nanogels, which are just like chemically crosslinked PNIPAM nano-zones (Fig. 1e). The PNIPAM nanogels show significantly thermo-responsive volume phase transition behaviours (Fig. 2a, Supplementary Fig. S1). All the interconnected microporous structures, the long chain structure of the polymer chains especially the dangling chains and the responsive nanogels, contribute to the thermo-responsiveness of NSG hydrogels cooperatively (Fig. 1f).

Our NSGs all have much faster response rate than that of NG (Fig. 4d,e). In our NSGs, the responsive nanogels, the flexible linear PNIPAM bridge chains between the nanogels as well as some linear grafting PNIPAM chains on the nanogels and the interconnected microporous structure are all beneficial to the rapid response rate. It is well known that the response rate of chemically crosslinked hydrogels is inversely proportional to the square of the gel dimension^34^. Upon increasing temperature across the LCST, the PNIPAM nanogels shrink very fast due to their nano size, and the flexible linear long PNIPAM bridge chains between the nanogels also shrink very fast because no crosslinked restriction exists for their response. Furthermore, the linear grafting PNIPAM chains on the nanogels shrink even faster, because they have freely mobile ends^35^. During the rapid shrinking, the linear grafting PNIPAM chains shrink and aggregate to shrunken ANG nanogels, and leave interconnected microporous spaces for water escaping from the hydrogels (Fig. 1f). The SEM images show that freeze-dried NSG hydrogels are of porous structures (Fig. 3), which indicates that interconnected channels exist in the NSG hydrogels for the fast transport of water. On increasing the polymerization time of ANG nanogels, the crosslinkage of NSG hydrogels decreases; as a result, the thermo-responsive shrinking rate becomes faster (Fig. 4d). On increasing the NIPAM concentration, the linear PNIPAM bridge chains connecting the nanogels become longer and more flexible; as a result, the thermo-responsive shrinking rate also becomes more rapid (Fig. 4e). For the NSG hydrogel NSG40-1.5, the characteristic time of hydrogel shrinking from fully swollen state to fully collapsed state for a hydrogel sample with characteristic dimension of >22 mm is only ~6 min, which is much faster than that of NG2-1.5, and also faster than that of previous nano-clay-composite hydrogels (typically >10 min for a cylinder hydrogel sample with characteristic diameter of 5.5 mm)^21^,^22^,^36^.

### Elastic characteristics of nano-structured smart hydrogels

**Figure 5:**
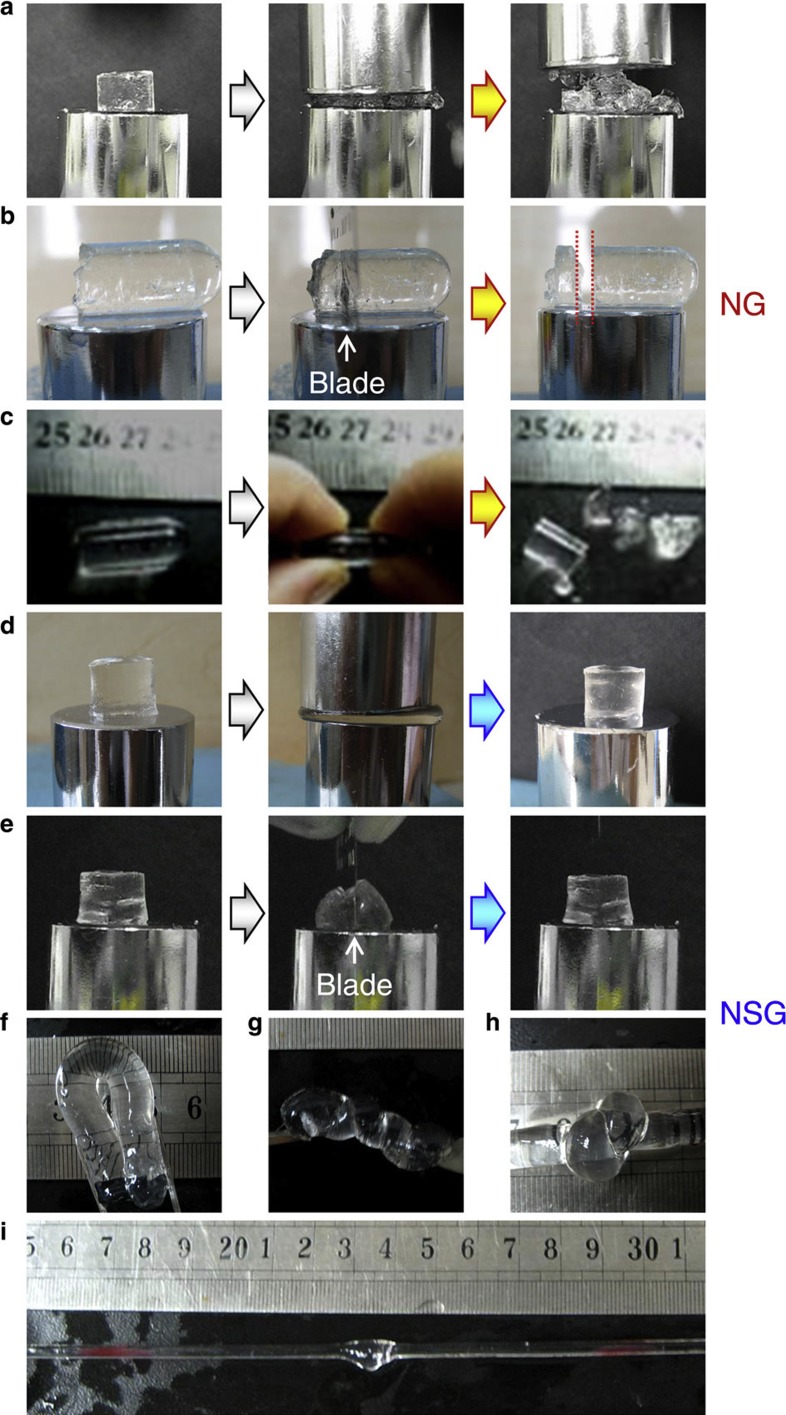
The NSGs are elastic and stretchable. (**a**–**c**) Optical images showing that NG2-1.5 samples are too brittle to sustain compression (**a**, Supplementary Movie 1), slicing with a blade (**b**) or elongation (**c**). (**d**–**i**) Optical images showing that NSG (NSG20-1.5-0.5C) samples are very elastic to sustain a high compression (**d**, Supplementary Movie 2), resist slicing with a blade (**e**, Supplementary Movie 3), and withstand high level of deformations such as bending (**f**), twisting (**g**), knotting (**h**) and extensive stretching (**i**). *T*=25 °C.

Our NSGs exhibit excellent elastic properties (Fig. 5). NG2-1.5 samples are too brittle to sustain compression (Fig. 5a, Supplementary Movie 1), slicing (Fig. 5b) or elongation (Fig. 5c). However, our NSG hydrogels are highly elastic and stretchable, behaving like rubbers. At 25 °C, the NSG hydrogel samples are very elastic to withstand high compression (Fig. 5d, Supplementary Movie 2), slicing with a blade (Fig. 5e, Supplementary Movie 3), or high level of deformations such as bending (Fig. 5f), twisting (Fig. 5g), knotting (Fig. 5h) and extensive stretching (Fig. 5i, Supplementary Movie 4). From the proposed nano-structure (Fig. 1), flexible linear PNIPAM bridge chains connecting nanogels are assumed to have relatively uniform and large length, and they exhibit a natural coil state if no load is applied. They could be elongated under stress until they become straight, and can recover to the lowest energy coil state when the stress is taken away. Thus, the stress can be dissipated effectively to the large number of flexible PNIPAM bridge chains connecting nanogels in the NSG hydrogels. So, the NSG hydrogels exhibit highly elastic and stretchable properties.

**Figure 6:**
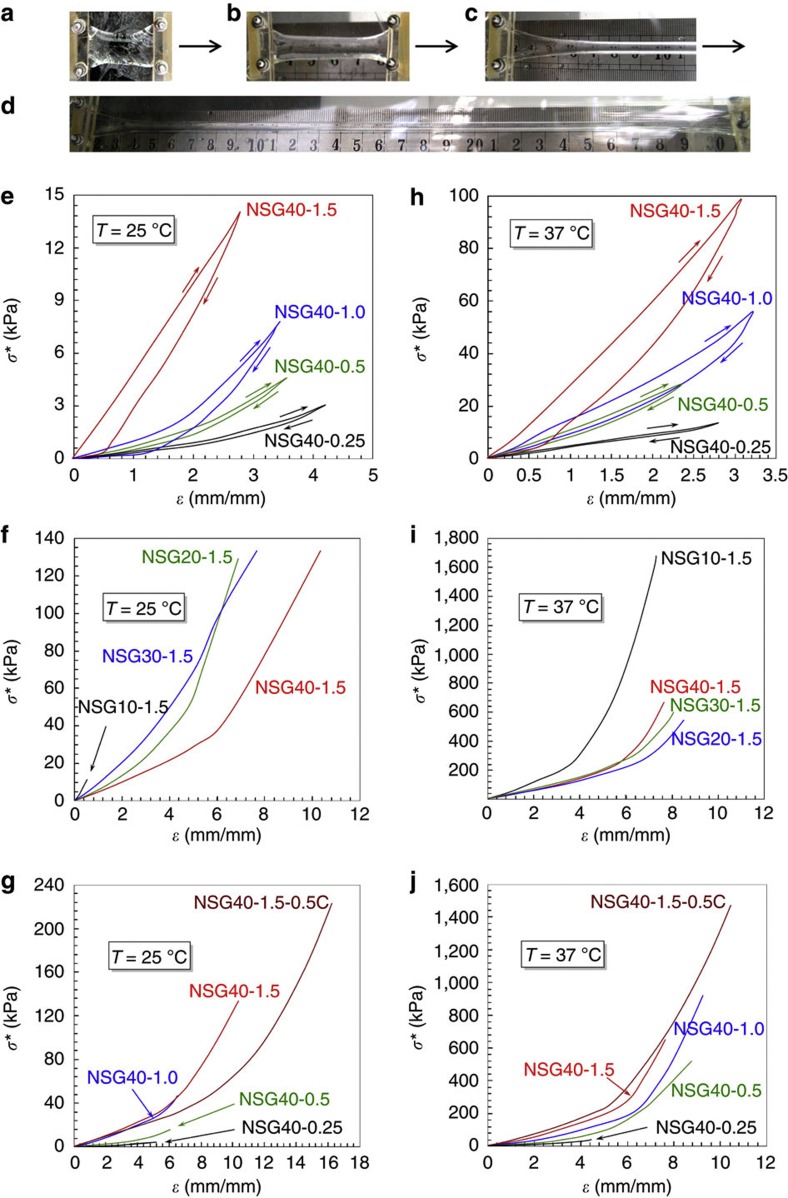
Elastic behaviours of NSGs. (**a**–**d**) Optical images showing the process of a NSG hydrogel being stretched to ~12 times its initial length in a tensile machine. (**e**–**j**) Typical stress–strain curves of NSG hydrogels of various compositions at room temperature (25 °C, <LCST; **e**–**g**) and at body temperature (37 °C, >LCST; **h**–**j**), in which σ* is the tensile stress and *ε* is the tensile strain. (**e**–**h**) The hydrogels are each loaded to a certain stretch before rupture and then unloaded. (**f**,**g**,**i**,**j**), The hydrogels are pulled to rupture in each test.

**Figure 7:**
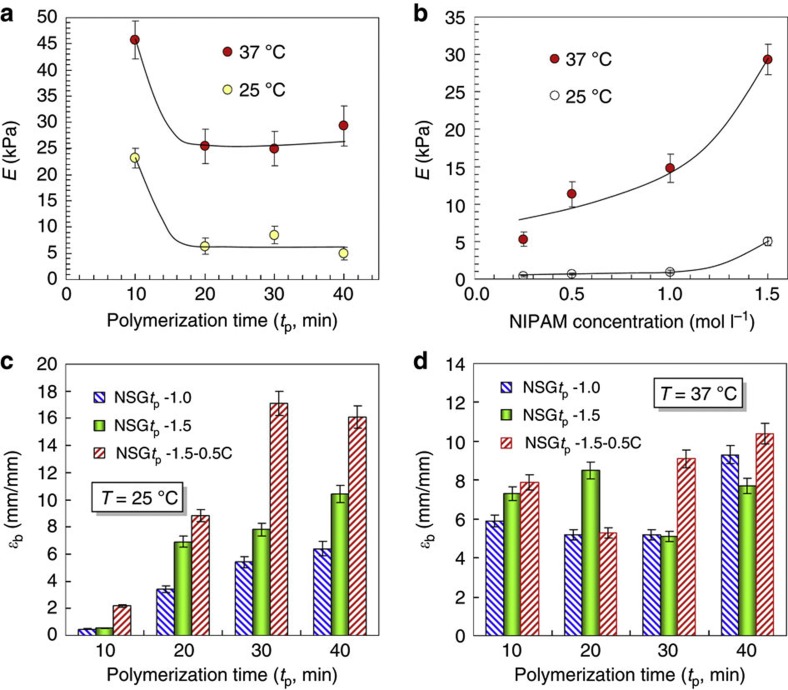
Young’s modulus and elongation ratio at break of NSG hydrogels. (**a**,**b**) Effects of ANG polymerization time (**a**, NIPAM concentration is fixed as 1.5 mol l^−1^) and NIPAM monomer concentration (**b**, *t*_p_=40 min) on the Young’s moduli (*E*) of NSG hydrogels at the tensile strain of 50%. (**c**,**d**), Effects of ANG polymerization time, NIPAM monomer concentration and ANG nanogel concentration on the elongation ratio at break (*ε*_b_) of NSG hydrogels of various compositions at room temperature (**c**) and body temperature (**d**).

The composition and environmental temperature greatly affect the elastic properties of our NSGs (Fig. 6). Mechanical tensile-stress experiments of NSG hydrogel samples are performed using a tensile machine. The NSG hydrogel sample is fixed by two plexiglass clamps (Fig. 6a–d), and the stretch rate is 10 mm min^−1^ in both loading and unloading. The tensile experiments are performed at both room temperature (25 °C, <LCST) and body temperature (37 °C, >LCST). On increasing the NIPAM concentration but keeping the polymerization time of ANG nanogels constant, the PNIPAM bridge chains connecting the nanogels become longer as mentioned above; as a result, the NSG hydrogels dissipate energy more effectively, as shown by the increased hysteresis in the stress–strain curves (Fig. 6e,h). For each hydrogel at the tensile strain of 50%, the Young’s modulus at 37 °C is much larger than that at 25 °C (Fig. 7a,b, Supplementary Fig. S3). On increasing the polymerization time of ANG nanogels from 10 min to 20 min, the Young’s modulus of NSG hydrogel decreases significantly (Fig. 7a). On increasing the NIPAM concentration, the density of polymeric networks inside the NSG hydrogels increases; as a result, the Young’s moduli of NSG hydrogels increase (Fig. 7b). On decreasing the ANG nanogel content, the Young’s modulus of NSG hydrogel decreases slightly at 25 °C (Supplementary Fig. S3a), but nearly does not change at 37 °C (Supplementary Fig. S3b). NSG hydrogels prepared with less ANG nanogels, are less crosslinked in structure and then more flexible in stretching; therefore, the Young’s moduli are smaller at 25 °C. At 37 °C, the long PNIPAM bridge chains between the nanogels shrink easily and become hydrophobic meanwhile, and then entangle with each other and form reversible additional physical crosslinking, which could offset the effect from less crosslinking and result in a nearly unchanged Young’s modulus.

At 25 °C, the elongation ratio at break of our NSG hydrogels can be as large as 1,710% (Fig. 7c), which is even larger than that of the nano-clay-composite PNIPAM hydrogels (typically <1,430%)^21^,^22^ and microgel-reinforced hydrogels (typically <1,270%)^29^. As the elastic nanogels can also contribute to the elastic property of the NSG hydrogels but the solid nano-clays cannot contribute to the elastic property of hydrogels, it is reasonable that the elongation ratio at break of our NSG hydrogels is larger than that of nano-clay-composite PNIPAM hydrogels. On increasing the polymerization time of ANG nanogels, the elongation ratio at break of NSG hydrogel increases (Figs 6f, 7c). It has been reported that the entanglement between polymer chains and networks inside a hydrogel can significantly increase the mechanical properties of the hydrogel^37^. Therefore, the NSG hydrogels become more elastic and stretchable on increasing the polymerization time of ANG nanogels, mainly due to the stronger entanglement of polymer chains between the ANG nanogels. The elongation ratio at break of NSG hydrogel also increases on increasing the NIPAM concentration (Figs 6g, 7c). When the NIPAM concentration is larger, longer and more flexible PNIPAM bridge chains are generated connecting the nanogels; as a result, the NSG hydrogels are more elastic with larger elongation ratio at break as well as higher true tensile strength (Fig. 6g, Supplementary Fig. S4a). On decreasing the ANG nanogel content but keeping high concentration of NIPAM, both larger elongation ratio at break and higher true tensile strength are obtained for the NSG hydrogels (Figs 6g, 7c, Supplementary Fig. S4a), due to the less crosslinked structure and the longer flexible PNIPAM bridge chains.

At 37 °C, the elongation ratio at break still remains high but the true tensile strength of NSG hydrogels increases significantly than that at 25 °C. At 37 °C, additional and reversible physical crosslinking points form due to the hydrophobic interaction between the adjacent shrunken PNIPAM bridge chains, which results in the increase of true tensile strength of NSG hydrogels as well as some interesting phenomena. Unlike the case at 25 °C, the polymerization time of ANG nanogels does not significantly affect the elongation ratio at break of NSG hydrogels at 37 °C (Figs 6i, 7d). At 37 °C, both the nanogel crosslinkers and the bridge PNIPAM chains between nanogels are in the shrunken and hydrophobic state, and the hydrophobic interaction and aggregation of long-bridge PNIPAM chains between nanogels result in large amount of ‘pseudo’ physical crosslinking points. Therefore, the ‘virtual’ crosslinking degrees inside the NSG hydrogels, that is, the sum of chemical crosslinking degree and ‘pseudo’ physical crosslinking degree, are almost the same no matter how long is the polymerization time of ANG nanogels. When the polymerization time of ANG nanogels is relatively long, for example, 30 or 40 min, both the elongation ratio at break and the true tensile strength of NSG hydrogels at 37 °C increase significantly on decreasing the ANG nanogel content (Figs 6j, 7d, Supplementary Fig. S4b). When the content of ANG nanogel crosslinkers is less, the PNIPAM bridge chains between nanogels become longer inside the NSG hydrogels. The longer the PNIPAM bridge chains between nanogels, the more the additional and reversible ‘pseudo’ physical crosslinking points form inside the NSG hydrogels at 37 °C. As a result, both the elongation ratio at break and true tensile strength of NSG hydrogels are improved to some extents. Unexpectedly, when the NSG hydrogels are prepared with ANG10 nanogels and high NIPAM concentration such as 1.5 mol l^−1^, the NSG10-1.5 and NSG10-1.5-0.5C hydrogels at 37 °C have extraordinary true tensile strength (Fig. 6i, Supplementary Fig. S4b), mainly due to both the densely chemical crosslinking existing in the hydrogels and the densely ‘pseudo’ physical crosslinking from the densely packed polymeric networks. The elastic properties of smart hydrogels at body temperature are demonstrated for the first time in this study, and the results show that they are significantly different from that at room temperature, which provides valuable information for various applications of smart hydrogels.

## Discussion

We have demonstrated simple and controllable synthesis of a novel type of nano-structured smart hydrogels with large responsive swelling ratio, rapid response rate and high elasticity, by constructing the nano-structured architecture with activated responsive nanogels as nano-crosslinkers. The generated NSGs are heterogeneous 3D networks composed of responsive polymer chains crosslinked by responsive nanogels, which are just like chemically crosslinked responsive nano-zones. With the proposed nano-structured architecture, factors conducive to improving both stimuli-responsive and elastic properties are simultaneously introduced into the smart hydrogels. The stimuli-responsive and elastic properties of the NSGs can be easily customized by changing the polymerization time of responsive nanogels, the monomer concentration or the nanogel content. By using a proper recipe, a nano-structured smart hydrogel could have all the large responsive swelling ratio, rapid response rate and high elasticity. Such a combination of large responsive swelling ratio, rapid response rate and high elasticity, along with an easy method of fabrication, makes the nano-structured smart hydrogels ideal candidates for further investigations and applications. As the cytocompatibility of PNIPAM hydrogels have been confirmed by a lot of previously reported investigations^38^,^39^,^40^, our nano-structured thermo-responsive PNIPAM hydrogels exhibit promising application prospects in biological and biomedical fields. The strategy of the nano-structured architecture and the simple synthesis procedure presented here circumvent the difficulties in simultaneously improving stimuli-responsive and elastic properties of smart hydrogels. It can be used to fabricate various kinds of responsive or even non-responsive hydrogels with satisfactory performances for numerous applications^1^,^2^,^3^,^4^,^5^,^6^,^7^,^8^,^9^,^10^,^11^ just by using other monomer materials, which might be a fertile area of research. Owing to the excellent concurrent stimuli-responsive and elastic properties, the nano-structured smart hydrogels will provide ever better performances in their myriad applications including micro-actuators, drug delivery systems, artificial muscles and cartilages, scaffolds for tissue engineering and matrices for bioseparation, and may open up new fields of application for smart hydrogels.

## Methods

### Fabrication of ANGs

ANGs bearing unsaturated double bonds were fabricated by precipitation polymerization of NIPAM and MBA, which is initiated by KPS in the presence of SDS. First, 50 ml of pure water in a round-bottom flask was bubbled with N_2_ gas for 5 min to remove the dissolved oxygen. Then, 1.13 g NIPAM (10 mmol), 0.0616, g MBA (0.4 mmol) and 0.027 g KPS were added to the water and mixed by a magnetic bar until total solution. Next, 0.27 g SDS was added to the water and further mixed by a magnetic bar until total solution. N_2_ gas was blown into the flask above the liquid level for additional 5 min and then the flask was sealed immediately. The polymerization was performed for 10, 20, 30, 40 and 60 min at 60 °C with stirring at about 300 r.p.m. The polymerization was terminated by cooling the ANG dispersions immediately in an ice-water bath. The ANG nanogel dispersions were then stored at 0 °C for subsequent use.

The quantitative concentrations of unsaturated double bonds in the ANG dispersions prepared with different polymerization times were obtained with the aqueous bromide-bromate reagent method^41^. To make sure the unsaturated double bonds exist on the ANG nanogels, hydroquinone was added into the ANG nanogel dispersions as polymerization inhibitor immediately after the polymerization, and then the ANG nanogels were dialyzed against pure water using dialysis bags with a molecular weight cutoff of 8,000–14,000. The dialysis was performed for >1 week with refreshing water twice a day, and then the ANG nanogels were freeze-dried for 48 h. The chemical compositions of the freeze-dried ANG nanogels were then analysed by a FTIR spectroscope with a KBr method (IR Prestige-21, Shimazu). The hydrodynamic diameters of ANG nanogels in water at temperatures ranging from 25 to 50 °C were measured by dynamic light scattering (Zetasizer Nano ZS90, Malvern) equipped with a He-Ne light source (*k*=633 nm, 4.0 mW). Before each data collection, the highly diluted ANG nanogel dispersion in deionized water was allowed to equilibrate for 20 min at each temperature. Transmission electron microscope (TEM, Tecnai G2 F20 S-TWIN, FEI) was employed to study the morphology of nanogels in dry state. For TEM observation, the thoroughly dialyzed ANG nanogel dispersions were first diluted in ethanol and ultrasonicated for 5 min, and then dripped onto a copper grid covered with a perforated carbon film and subsequently dried at room temperature.

### Preparation of hydrogels with nano-structured architecture

NSGs were prepared from the ANGs and NIPAM monomer with TEMED as an accelerator at 0 °C. Typically, a mixture of 4.5 ml above-fabricated ANG nanogel dispersion and 0.5 ml pure water was bubbled with N_2_ gas for 5 min to remove the dissolved oxygen in a glass test tube, which was immersed in an ice-water bath. Then, a certain amount of NIPAM was dissolved in the mixture. Next, 20 μl of TEMED was injected into the test tube. The test tube was then sealed immediately, and kept stilly in the ice-water bath for 2 h and then at room temperature for 48 h. In the experiments, the NIPAM molar concentration in the mixture solution was typically 1.5, 1.0, 0.5 and 0.25 mol l^−1^, and the correspondingly fabricated NSG hydrogels were coded as NSG*t*_p_-1.5, NSG*t*_p_-1.0, NSG*t*_p_-0.5 and NSG*t*_p_-0.25 with the NIPAM molar concentration as the suffix and ‘*t*_p_’ indicating the polymerization time for ANG nanogel preparation (Table 1). To investigate the effect of ANG nanogel content on the NSG hydrogel performance, NSG hydrogels were also prepared with a mixture of 2.5 ml ANG nanogel dispersion and 2.5 ml pure water instead of the above-mentioned mixture of 4.5 ml ANG nanogel dispersion and 0.5 ml pure water, and ‘0.5C’ was added to the code of the NSG hydrogels as the suffix.

The chemical compositions of the hydrogels were also analysed by FT-IR. SEM (JSM-5900LV, JEOL) was employed to study the microstructures of hydrogels in freeze-dried state. To prepare freeze-dried samples, hydrogels were transferred into liquid nitrogen for 15 min after they had been maintained in 15 °C water for a week, and then freeze-dried at −43 °C under a vacuum of 0.1 Pa for 48 h to thoroughly remove the water. To observe the cross-sections, freeze-dried hydrogel samples were put into liquid nitrogen for a sufficient length of time, fractured mechanically and stuck to the sample holder. All the samples were sputter-coated with gold for 40 s before observation.

### Preparation of NG as control sample

NG was also prepared as the control sample according to the above-mentioned method but without any ANG nanogels. Pure water (5 ml) was used as the solvent, and the NIPAM molar concentration was 1.5 mol l^−1^ (0.8475, g, 7.5 mmol). To prepare the normal crosslinked hydrogel, MBA (0.0232, g, 0.15 mmol) was used as a crosslinker, and KPS (0.0135, g, 0.05 mmol) and 20 μl TEMED were added to initiate the polymerization. The fabricated NG was coded as NG2-1.5.

### Thermo-responsive property testing

To investigate the temperature dependence of equilibrium volume phase transition, NSG and NG hydrogels were cut into discs with thickness of 10 mm. The hydrogel discs were washed by excess of pure water for >1 week, and the water was refreshed twice a day. Then, the hydrogel discs were immerged in sufficient pure water in a transparent glass container with a calibrated scale. The container was placed in a thermostatic water bath with temperature fluctuation of ±0.1 °C. The equilibrium volumes of hydrogels at selected temperatures were recorded in the range from 15 to 45 °C. As the LCST of PNIPAM is ~32 °C (ref. 42), the test temperature range was selected to cross the LCST. At each temperature, the temperature was maintained constant for 8 h to ensure that the hydrogel samples have reached the equilibrium state before measurement. The diameters of hydrogel discs were recorded by a digital camera (PC1193, Canon) and surveyed by the calibrated scales. The equilibrium swelling ratio at *T* °C is defined as *V*_T_/*V*_45_=(*d*_T_/*d*_45_)^3^ or *V*_T_/*V*_p_=(*d*_T_/*d*_p_)^3^, where *V*_T_ and *V*_45_ are the equilibrium volumes of hydrogel at *T* °C and 45 °C respectively, and *V*_p_ is the initial volume of hydrogel in the preparation tube; *d*_T_ and *d*_45_ are the diameters of hydrogel at *T* °C and 45 °C, respectively, and *d*_p_ is the initial diameter of hydrogel in the preparation tube.

Dynamic thermo-responsive deswelling behaviours of hydrogels were measured by recording videos of the volume change processes by a digital camera after the environmental temperature jumped abruptly from 25 °C to 55 °C. The hydrogel samples, which have been equilibrated in cool water at 25 °C for >8 h, were suddenly transferred from the cool water at 25 °C into hot water at 55 °C. At the same time, the video record started. The dimensions of hydrogels were measured from the digital pictures intercepted from the movies. The deswelling rate of the hydrogel is expressed as *V*_t_/*V*_0_ as a function of time, in which *V*_t_ and *V*_0_ are the volumes of hydrogel samples at time *t* and at the beginning (*t*=0, equilibrated in water at 25 °C), respectively.

For each thermo-responsive test, at least five specimens were tested and the averages were obtained with s.d. for each data point.

### Elastic property testing

Mechanical tensile-stress experiments of NSG hydrogels were performed using a tensile machine at a stretch rate of 10 mm min^−1^. The water contents of the NSG hydrogels for elastic property tests were: 80.9% for NSG*t*_p_-1.5, 81.9% for NSG*t*_p_-1.5-0.5C, 86.5% for NSG*t*_p_-1.0, 92.2% for NSG*t*_p_-0.5, and 95.0% for NSG*t*_p_-0.25. The tensile experiments were performed at both 25 °C and 37 °C. The tensile stress, σ*, is defined as the force applied on the deformed hydrogel (*F*) divided by the real-time characteristic cross-sectional area of the deformed hydrogel (*A*), that is, σ*=*F*/*A*, in which *A*=*A*_0_* × L*_0_/*L* from the fact of volume constant, and *A*_0_ is the initial cross-sectional area of the hydrogel, *L*_0_ is the initial gauge length of the hydrogel, and *L* is the real-time gauge length of the deformed hydrogel. The tensile strain, *ε*, is defined as the ratio of gauge length change (*ΔL*) to initial gauge length of the hydrogel, that is, *ε=ΔL*/*L*_0_=(*L*−*L*_0_)/*L*_0_. The Young’s modulus of the hydrogel at a certain tensile strain, *E*, is calculated by the slope of the stress–strain curve at the corresponding point, that is, E=dσ*/*dε*. The largest tensile stress at break of the NSG hydrogel is defined as the true tensile strength at break (*σ*_b_), and the largest tensile strain at break of the NSG hydrogel is defined as the elongation ratio at break (*ε*_b_). For each elastic property test, at least five specimens were tested and the averages were obtained with s.d. for each data point.

## Author contributions

L.-W.X. and L.-Y.C. conceived and designed the study. L.-Y.C. supervised the research. L.-W.X. performed the experiments. All authors discussed the results and contributed to the data interpretation. L.-W.X. and L.-Y.C. wrote the manuscript and all authors commented on the manuscript.

## Additional information

**How to cite this article**: Xia, L.-W. *et al.* Nano-structured smart hydrogels with rapid response and high elasticity. *Nat. Commun.* 4:2226 doi: 10.1038/ncomms3226 (2013).

## Supplementary Material

Supplementary Figures Supplementary Figures S1-S4

Supplementary Movie 1 The normal hydrogel (NG2-1.5) is very fragile and able to sustain a high compression at room temperature (25 °C)

Supplementary Movie 2 The nano-structured hydrogel (NSG20-1.5-0.5C) is very elastic and able to sustain a high compression at room temperature (25 °C).

Supplementary Movie 3 The nano-structured hydrogel (NSG20-1.5-0.5C) is very elastic and able to resist slicing with a blade at room temperature (25 °C).

Supplementary Movie 4 The nano-structured hydrogel (NSG20-1.5-0.5C) is very elastic and able to withstand a high level of stretching at room temperature (25 °C).

## Figures and Tables

**Table 1:** Chemical composition for preparation of NSG hydrogels.

**NSG hydrogel code**	**NIPAM**		**ANGt**_***p***_ **nanogel dispersion (ml)**	**Water (ml)**
	**Concentration (mol l^−1^)**	**Amount (g)**		
NSG*t*_p_-1.5	1.5	0.8475	4.5	0.5
NSG*t*_p_-1.0	1.0	0.5650	4.5	0.5
NSG*t*_p_-0.5	0.5	0.2825	4.5	0.5
NSG*t*_p_-0.25	0.25	0.1413	4.5	0.5
NSG*t*_p_-1.5-0.5C	1.5	0.8475	2.5	2.5

Note: the ‘*t*_p_’ in the code of NSG hydrogels and corresponding ANG nanogel dispersions refers to the polymerization time for the ANG nanogel preparation. In the NSG hydrogel preparation, 20 μl of TEMED is used in each case and the temperature is 0 °C.
