# Supplementary material for: Nano-structured smart hydrogels with rapid response and high elasticity
Source: Nat Commun. 2013 Jul 31;4:2226. doi: 10.1038/ncomms3226 (PMC3731657; doi:10.1038/ncomms3226)
Supplement: Supplementary Figures — S1-S4 [file ncomms3226-s1.pdf]

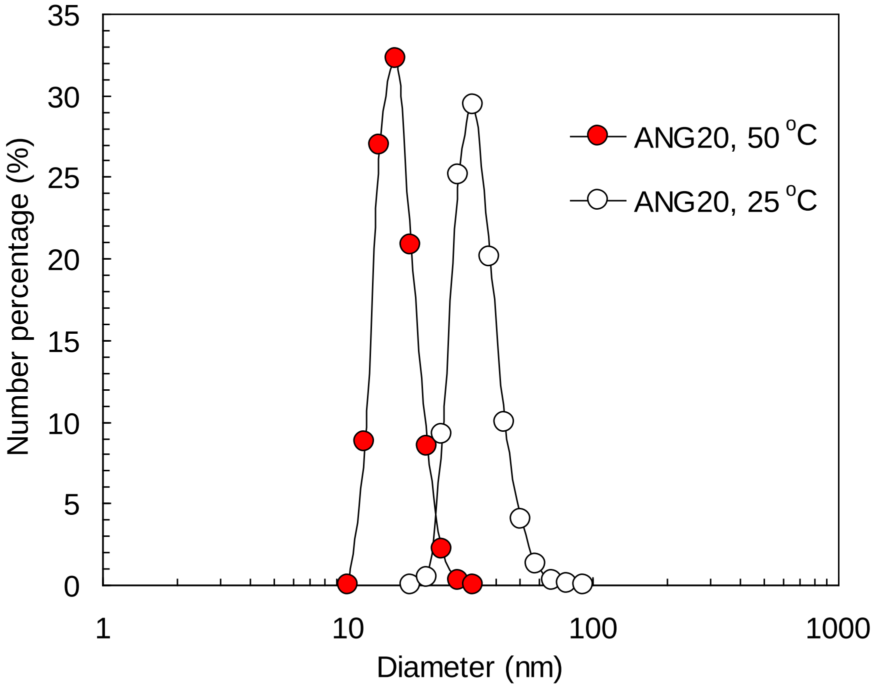

(a)

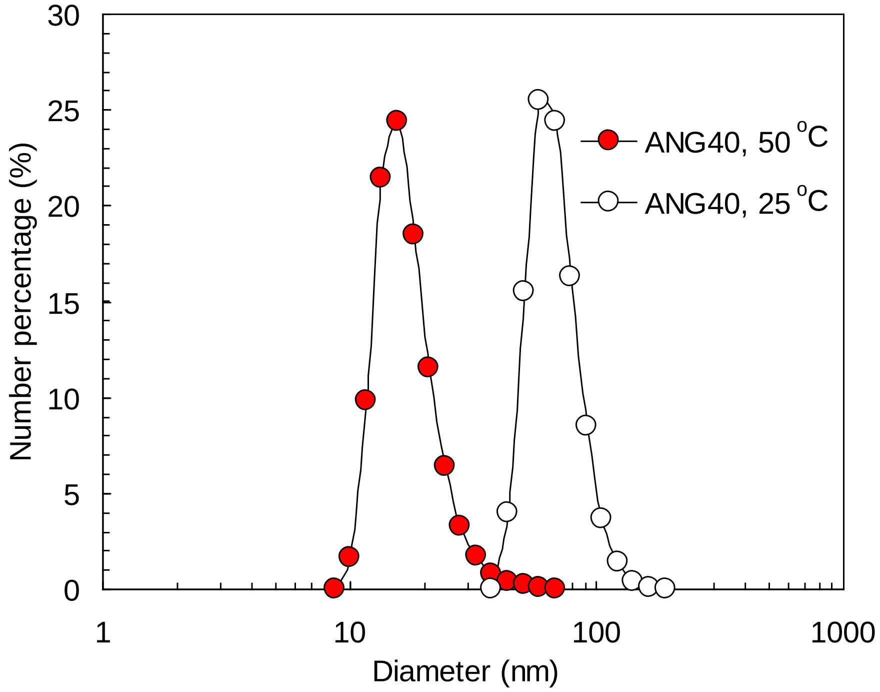

(b)

Supplementary Figure S1 │ Thermo-responsive hydrodynamic diameters of nanogels. Effects of polymerization time and temperature on the hydrodynamic diameters of ANG20 (**a**) and ANG40 (**b**) nanogels in water detected by dynamic light scattering.

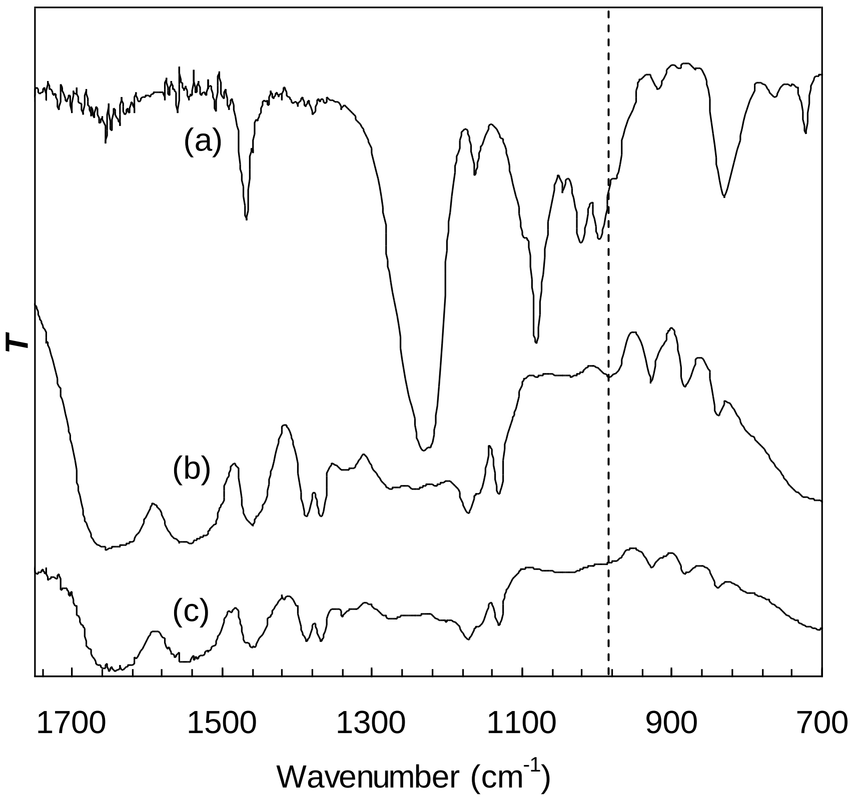

Supplementary Figure S2 │ Chemical confirmation of nanogels and NSG hydrogels. FT-IR spectra of surfactant SDS (a), activated nanogels ANG20 after thorough dialysis against water for 7 days (b), and freeze-dried NSG20-1.5 after fully washed by water (c). The peak at 981 cm^{-1} refers to unsaturated double bonds. SDS is also not found in either dialyzed ANG nanogels or washed NSG hydrogels. Unsaturated double bonds exist in thoroughly dialyzed ANG nanogels but not in NSG hydrogels.

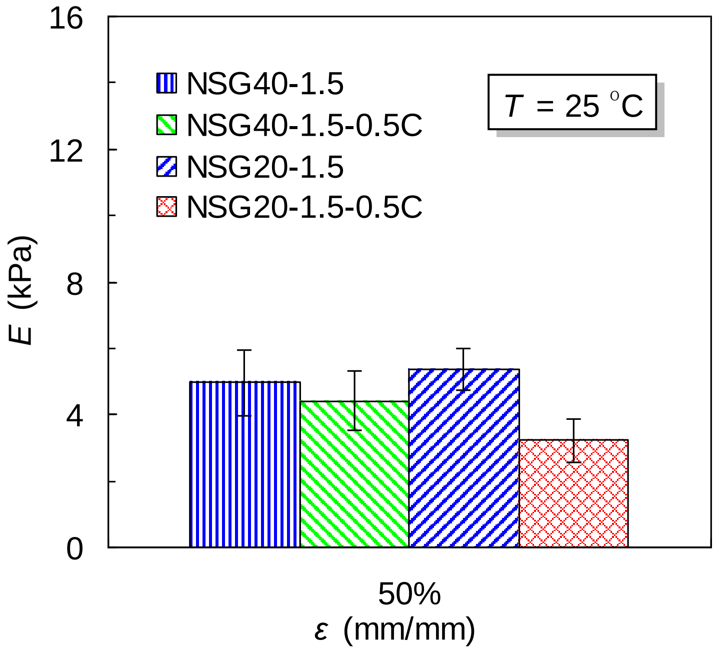

(a)

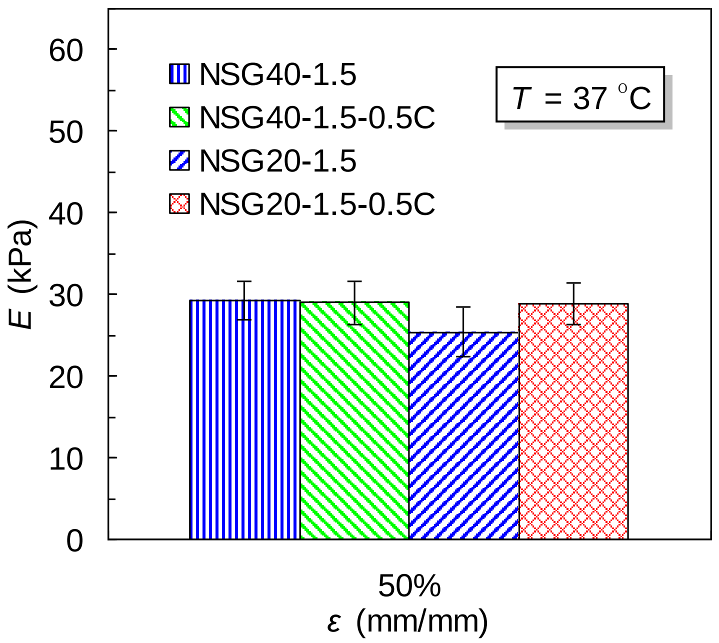

(b)

Supplementary Figure S3 │ Young's moduli of NSG hydrogels. Effects of the ANG nanogel concentration on the Young's moduli of NSG hydrogels at the tensile strain of 50% at room temperature (25 °C, below the LCST) (a) and at body temperature (37 °C, above the LCST) (b).

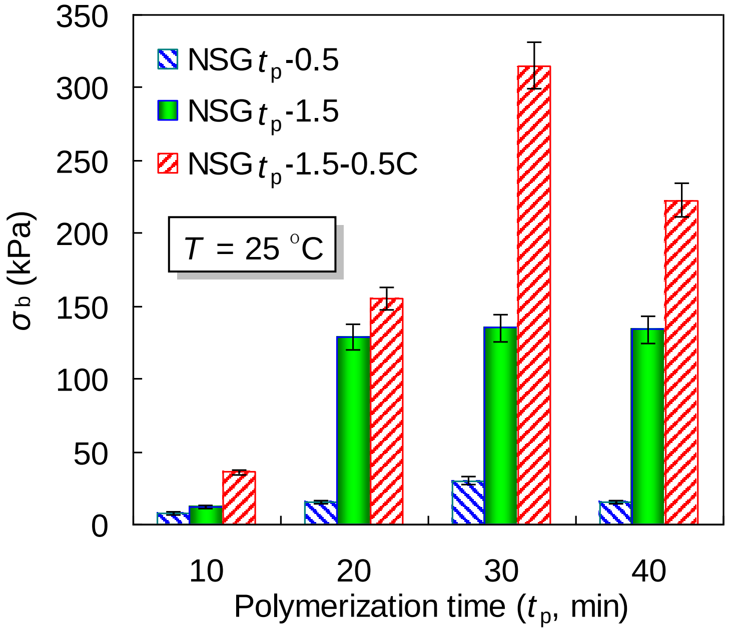

(a)

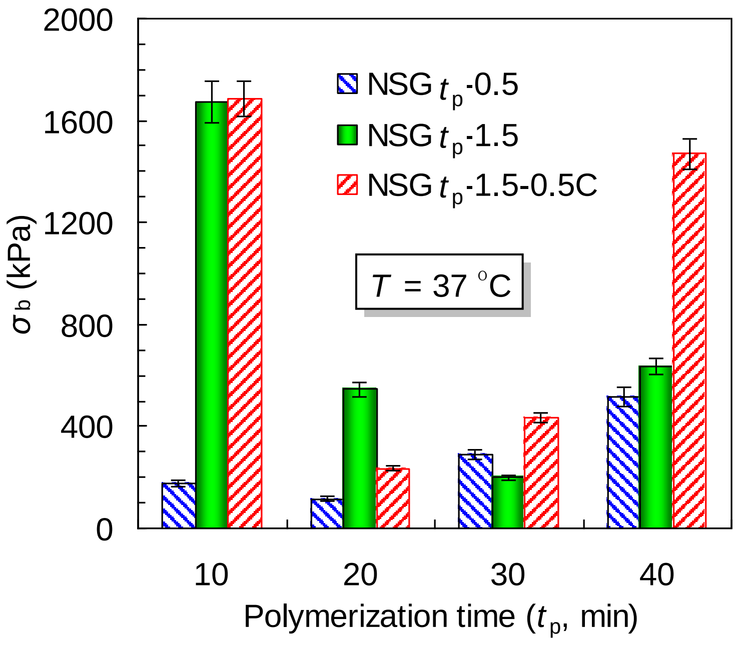

(b)

Supplementary Figure S4 │ True tensile strength at break of NSG hydrogels. Effects of the ANG polymerization time, NIPAM monomer concentration and ANG nanogel concentration on the true tensile strength at break of NSG hydrogels of various compositions at room temperature (25 °C, below the LCST) (a) and at body temperature (37 °C, above the LCST) (b).
